# On Surgical Education

**Published:** 1819-05

**Authors:** Barnard Van Owen


THE LONDON

### Medical and Physical Journal.

5 OF VOL. XLI.] MAY, 1819. [NO. 243.

"For many fortunate discoveries in medicine, and for the detection of numerous errors, the world is indebted to the rapid circulation of Monthly Journals; and there never existed any work to which the Faculty in EUROPE and AMERICA were under deeper obligations than to the Medical and Physical Journal of London, now forming a long, but an invaluable, series."—RUSH.

*For the London Medical and Physical Journal.*

### *On Surgical Education;* by BARNARD VAN OWEN, Esq.

THE unrestrained freedom of intercourse between England and the continent has afforded such ample opportunities of investigating the scientific and literary institutions of other countries, that it is surprising we should have profited so little by the occasion; and, above all, that our system of surgical education should have remained unaltered, whilst that of our neighbours is in many respects so eminently superior to our own. This is the more remarkable, as the enlightened and philosophic spirit of the present age disclaims all the petty prejudices of national vanity, and seeks only the advancement of science and the amelioration of human nature: I am, therefore, induced to believe, that many of the excellencies of the French and German schools are but imperfectly understood, and that I am performing a task not wholly uninteresting to the profession, or useless to the public, in endeavouring to point out those parts of the foreign systems which may deserve imitation; for, whilst it must be confessed that London is an admirable school of anatomy and surgery, and that the number and extent of its hospitals, the great ability of the professors, and the facilities of dissection, offer to the industrious student opportunities of improvement scarcely any-where else to be found,—it is equally undeniable that the negligent are too much allowed to waste those opportunities; that the important offices of dresser and house-surgeon are too often filled by the ignorant and inexperienced; and that the diploma of the Royal College is too often bestowed on men very inadequately fitted to discharge the duties of the profession. The only stimulus given to the London student is that of emulation: minds, panting with scientific ardour and anxious to excel, find ample opportunities of research; and hence the number of clever young men to be met with in our hospitals, and of great surgeons in our cities. But this is not enough: we should take care that the public health be never intrusted to licensed ignorance, who, having lounged through the wards of an hospital, attended a few lectures, and undergone a superficial examination, are enabled to denominate themselves surgeons and accoucheurs, and practise to the injury of their patients and the disgrace of their science and country. That this too often occurs in England is but too true, and it is equally true that it can scarcely ever occur in France or Germany; the period of study insisted on being much longer, and the examinations much more severe than here.

How frequently does it happen that a young man, after having spent four or five years, as an apothecary's apprentice, in the *very scientific employment* of mixing draughts and powders, is sent to London to devote *one* twelve-month to the acquirement of all the sciences connected with medicine, surgery, and midwifery. Often not content with this Herculean labour, and wishing to initiate himself in practice, he procures a dressership at some hospital; and, ignorant and inexperienced as he may be, he is immediately entrusted with the care of the most important cases. It were a waste of time to point out the improprieties of such a system, or to trace the evils that may result from it when ignorance and self-sufficiency chance to be united: suffice it to say, that this can never occur in France. The students there form three classes:—1st, such as only follow the surgeons during their visits to the wards, and observe what is done; 2nd, the *eléves externes*, who prepare the dressings and bandages, and assist in applying them; and, 3rd, the *eléves internes*, who are somewhat analogous to our house-surgeons. These classes vary in importance, but the distinction is not acquired, as with us, by dint of money, but by attentive study and observation. The eléves externes are selected from the mass of students by strict examination, and the eléves internes from them by a similar process; so that those only can be elevated to superior rank who can display superior ability, and the patients are entrusted only to the skilful and well-informed. Having thus finished his academic studies, the young Frenchman is entitled to demand his examinations: they are five in number, embrace all the sciences connected with our profession, and two are entirely in Latin. It is true that his attendance at the lectures and hospitals is as optional as here; but, when the strictness of the examinations is remembered, it would be vain to imagine that such attendance can be neglected. Although it may appear that nothing can be superior to this system of education, yet it is by no means free from faults; but is in this evidently superior to our own, that it seems to secure the public against regular but ill-qualified practitioners.

One of the great excellencies of foreign hospitals is the clinical lectures, constantly delivered; and it is almost a paradox, that, whilst those of Edinburgh are so much lauded, similar ones are so very rarely delivered in London. There cannot be a doubt that nothing contributes more to the advancement of the student, and to the giving him clear and distinct ideas of diagnosis, than such lectures; yet it must be remembered that they prevent, in some measure, the free exercise of his judgment: for how can he unbiassed examine a case, of the nature of which he is previously informed? To render these lectures, therefore, truly useful, they should only be delivered on a small number of select cases, which may serve as examples for the examination of others; and the following plan, pursued in some of the schools of Germany, should be adopted:—The students are there divided into two classes; those who only observe, and those who practise. The former see the cases, hear the reports, and attend the lectures; the latter have cases given to their care by the professor, to treat accordingly to their own judgment, subject to the observation of their teacher; and all are thus gradually introduced into active practice, in a manner which, whilst it gives full scope to their talents, prevents their inexperience proving hurtful. In this way, too, they are often allowed to perform operations under the directing eye of their master, and the critical regard of their fellow pupils.

The students of Paris, as well as those of most of the German schools, possess the advantage of a good medical library and museum. The excellence of the public libraries abroad, and the ease of access to them, is highly praiseworthy, and must prove inestimable to the student. To regard these noble depositaries of science creates in the mind of the English surgeon a degree of regret, mingled with a feeling of shame, that the only public library in London, that of the British Museum, is entered with some difficulty, and is rather suited to antiquarian research than to the purposes of general study; and it is least of all a medical library, which must be regarded as a great desideratum in this metropolis, but imperfectly supplied by the private collections of hospitals and societies.

Throughout the continent, I believe, nearly the same course of study must be pursued, and nearly the same probations undergone, whether a degree in medicine or surgery be desired. Such an arrangement cannot be too highly praised, for it is quite impossible entirely to separate these two branches of the profession, either in theory or practice: yet, until very lately, how little was the theory or practice of medicine studied here except by regular physicians? The Company of Apothecaries are doing much to remedy this evil, and their exertions promise to procure for the public a set of well-educated practitioners; but the practice of midwifery is still uncontrolled; and but too many are the fatal instances on record of the misery produced by the union of audacity and ignorance.

Having thus enumerated some of the faults in the mode of education adopted in England, and displayed some of the chief excellencies of the French and German schools, it may fairly be asked of me, how it arises that London possesses at this moment a greater number of truly celebrated men than any other city; so that what most astonished M. Roux, on his late visit to this metropolis, was the many persons who (to use his own phrase) are the representatives of surgery.

In France, lectures on every branch of medical and philosophical science, together with excellent museums and libraries, are gratuitously open to the student; the hospitals and clinical courses may also be attended without any expence: whilst, in London, the student is deprived of some of these advantages, and the expence necessarily incurred prevents his hearing the lessons or attending the practice of more than one profession. How, then, does it arise, that the number of eminent surgeons is so much greater here? What is there wanting in the French system, or what the peculiar excellence of our own, which serves to overbalance its faults? This question is difficult of solution; and I venture to throw out those few ideas which have occurred to me, not with a hope of solving the paradox, but from a wish to attract the attention of some one better able to elucidate the subject. I am inclined to regard the gratuitous mode of education pursued in France as a great evil, and the chief reason why truly great men are more rare there than here. The pupils who crowd the hospitals of Paris are mostly of the poorer and lower orders, devoid of the manners of gentlemen, and ignorant of the habits of good society. It is surely not to be expected that, amongst such men, many should be found anxious to acquire celebrity, or who bring to the study of the profession minds stored with the principles of philosophic science, or sensible of the importance and dignity of their pursuits. Emulation is a passion peculiar, I believe, to great, or at least to cultivated, minds, and is not generally

4

found in men taken from the lower classes of the people: yet such are the majority of the French students; and from such what can be expected? They study a profession, by the practice of which they hope to earn a livelihood; and, having obtained their diploma, they are much more intent on the means of procuring a subsistence than on the advancement of science, or the acquisition of posthumous fame. To the same cause may be attributed the low degree of respectability attached to the profession in general at Paris. Should any one there attain, by his exertions, a high degree of eminence, he will, probably, amass a large fortune,—probably be ennobled by a title,—probably, by his writings, benefit mankind, and secure to himself a posthumous reputation; but a respectable rank in society he can scarcely hope for. Few, very few, of the medical men of that capital move in the higher circles; nor can it be otherwise, as neither the birth, education, or manners, of the majority fit them for refined society.

Besides all this, there is a radical fault in the mode of education in France, of a most important nature; one which is enough to counteract many advantages. The students of Paris are taught every thing, except to think and act for themselves; their memories, not their minds, are cultivated; they are the mere servants of their masters, and, therefore, become merely their imitators; and, as they do not usually read any modern language except their own, and foreign medical works are rather scarce in France, their ideas are not enlarged by contemplating various opinions. In the hospitals, the surgeons visit the patients daily, dress them, and apply all bandages; the eléves only spread the plasters, and assist in applying them: even the eléves internes, who have nearly finished their education, do no more than this. I well know that we carry the opposite practice too far, and that patients in our hospitals are frequently left too much to the care of pupils but ill fitted for their situations: but this is only a fault of regulation; their qualifications should be ascertained before their admission. Yet the result of leaving the patient partly to the care of the pupil,—of allowing him to execute the orders of the surgeon,—of permitting him to prescribe in the absence of his master, and even to perform secondary operations,—is attended with this excellent advantage, that he is thus taught to apply principles as he learns them: he becomes interested in each case, and, partly responsible for its success, his powers of mind are developed, and his judgment constantly called into action. He criticises freely the opinions and practices of his teachers, and leaves the schools, not a theorist and a mere observer new to practice and undecided in his opinions, but one already accustomed to judge and act, and feeling some confidence in his own powers. The French student, on the contrary, cannot be much interested in a case which he has nothing to do with, and on account of which his mind is not actively employed: he need not even reflect on its nature as he passes from the wards to the clinical lecture-room, where he hears its history, cause, &c. fully explained, and of course adopts the opinions of his teacher. Thus, then, the French system of education does not tend to produce great men, for it checks the growth of rising genius by opposing its exertion; yet, by the course of study adopted, and the strictness of the examinations, it effectually guards against the dangers arising from superficial acquirement, by making each student well acquainted with the prevailing principles. In London it is precisely the reverse; for, whilst the ignorant and ill-informed are too much allowed to trifle with their fellow-creatures, the high respectability of the profession, and the great consideration paid to men of talent, tend to excite in the minds of all classes a love of science, and an enthusiastic desire for its advancement.

In England, a medical man is a gentleman by profession; it is necessary he should be a gentleman in fortune and manners also; his company is sought in the best circles, and he is treated with a degree of respect proportionate to his celebrity. He does not often bear titles, but proudly considers the title of physician or surgeon superior to every other. Here, then, the whole system of education rests on the basis of emulation. The most attentive student is sure of receiving some mark of approbation, and men are excited to exertion by the knowledge that professional eminence is amongst the highest honours they can possess, and by an enthusiasm for the advancement and dignity of their profession, excited and kept alive by the consciousness that each man's rank in society, as well as his private fortune, depends almost entirely on his personal merit as a medical man and a gentleman. Almost every student who enters the profession brings to the study of it a mind filled with its importance, and delighted with its respectability; generally, too, a mind stored more or less with the preliminary sciences. He commonly belongs to the higher or middle ranks of society, and joins, to an admiration of his pursuits, a profound respect for his teachers. In such a mind nothing is more natural than a desire to excel. The anxious hope of acquiring a reputation fills his imagination, inspires him with courage to pursue his studies, and stimulates him to the greatest endeavours; he pants for fame, for reputation, because he well knows that honour, respect, rank, and fortune, are the consequences. How pleasing is it to observe the wards of our hospitals filled with visitors; to see men of independent fortune, and surgeons who have grown grey in practice, crowd round the sick bed or the operating couch, with the same restless anxiety as the student who arrived but yesterday; to see persons of all ranks and ages, philosophers and philanthropists, practitioners and pupils, equally desirous of acquiring information, equally ardent for the extension of human knowledge and the relief of human sufferings. This truly interesting scene struck M. Roux with astonishment and admiration, because, as he was constrained to confess, it is very rare to see the French practitioner revisit the wards of a hospital.

Such is the present state of medical education amongst our neighbours and ourselves; and, to any one who for a moment reflects on it, it must be evident that our own system is by far too lax, and therefore requires amendment. That it would be easy to amend our system, so as to render it far more perfect, I shall endeavour to show; and I hope that some of those men who are now rendering their names so conspicuous in the annals of humanity, will think this object not unworthy their regard.

I am far from proposing that our hospitals, like those of Paris, should be under the direction of one Board of Control, and the pupil be allowed to visit all indiscriminately. Such an arrangement destroys that independence of action so necessary to the free exertion of talent; and the young student, who sees too many modes of practice, does not acquire any one correctly, but becomes a timid undecided practitioner; yet, when his principles are formed,—when he has passed through the prescribed course of education, and received his diploma,—the wards of every hospital should be thrown open to his inspection. Why should not the surgeon who resides in the neighbourhood of a great hospital be allowed the opportunity of improving his experience from so good a source? It is true that strangers are allowed to see operations occasionally at most of the great hospitals: but operations are but seldom required, and therefore but secondarily important to the general class of practitioners. It is in sitting by the bedside, and watching the changes of disease, that he can gain the most important information, and perhaps learn to avoid those bloody performances which are more or less the opprobrium of surgery. Besides, a too constant attendance even on operating days would generally be noticed, and at best it is a matter of courtesy, which places the visitor in some degree under an obligation. This should not be the case: an accepted physician or surgeon should have a right to attend the practice of any hospital. It is not to be expected that gentlemen will, after having finished their education, enter again as pupils, and pay again the customary fees; but the advantages which might result to science from allowing them free admission to the wards are incalculable.

I am equally averse to rendering education gratuitous. To sustain the respectability of the profession, it is necessary that those who belong to it should be of a certain rank in society, and therefore it is right that it should be attended with considerable expence: indeed, I doubt whether the study of surgery, as at present conducted, be sufficiently expensive or not; but, be this as it may, the difference in rank amongst pupils should by no means depend on money, but on merit only. Every student, on entering, should pay the same fee without reference to time, and be allowed to be considered a pupil until he shall have received a diploma. At the commencement of every six months, dressers should be chosen from the students by public examination, and should not be subjected to any additional expence on account of holding the dressership; the original fee paid by all students being so arranged as equally to remunerate the surgeons. A surgical clinical ward should be established in every hospital, to receive select cases; for this ward, clinical dressers should be chosen, by examination, from those serving, or who shall have served, the office of dresser. They should not only keep a register of all cases treated by the surgeons, but should have cases given to their own care, to manage publicly under the observation of their master. Besides all this, much might be done by the Royal College of Surgeons towards the advancement of science. Instead of a short course of fifteen lectures on Surgery and Comparative Anatomy, regular courses should be delivered annually of a nature rather calculated for the advanced student than the tyro; as Surgical Anatomy, Physiology and Pathology, both human and comparative, Surgery, and Medical Surgery. I should add Midwifery, which is properly a branch of surgery, but unfortunately, although connected with both branches of the profession, is neglected by both Colleges; and no examinations occur, no regulations exist, to prevent women, at the most eventful periods of their lives, falling a sacrifice to ignorance or stupidity. The lectures should be gratuitous, as they should not by any means interfere with private lecturers; and, for the same reason, no certificate of attendance should be given; but, in order that they might be worthy the body in whose theatre they are delivered, the lecturers should receive such salaries as would render the post worth the acceptance of the most celebrated persons. The superb Hunterian Museum should be open daily to unrestrained examination; and a public library and reading-room should be added, where the student might freely resort to pursue his researches. If these additional advantages were allowed to pupils, something more might be expected from them, and each might be desired to compose a thesis previously to being examined; and the examinations themselves should surely be both more protracted and severe.

To carry plans like these into execution would, I hope, not be unworthy the Court of Assistants. In recommending them, I am actuated by a wish for the advancement of science, and a patriotic desire to render London the most perfect school of surgery in the world.

I have said little of medicine, because this metropolis is not, properly speaking, a school of medicine. It might, however, be well if the Royal College of Physicians possessed the power of conferring degrees in medicine, as that of Surgeons does in surgery; although it is pity that these branches of the profession cannot be combined, and the same course of study be pursued, and the same degree assumed, for medicine, surgery, and midwifery.

*Fenchurch-Buildings; Feb.* 20, 1819.

---

*For the London Medical and Physical Journal.*

In page 369, and the heads in the succeeding pages, for OWEN read OVEN.
— 370, line 5, for *ignorance* read *ignorami*.
— 372, — 28, for *profession* read *professor*.

